# Biochemical properties and oxalate‐degrading activity of oxalate decarboxylase from bacillus subtilis at neutral pH

**DOI:** 10.1002/iub.2027

**Published:** 2019-02-26

**Authors:** Carolina Conter, Elisa Oppici, Mirco Dindo, Luigia Rossi, Mauro Magnani, Barbara Cellini

**Affiliations:** ^1^ Department of Neurosciences Biomedicine and Movement Sciences, Section of Biological Chemistry, University of Verona Verona Italy; ^2^ Department of Experimental Medicine University of Perugia Perugia Italy; ^3^ Department of Biomolecular Sciences University of Urbino “Carlo Bo” Urbino Italy

**Keywords:** oxalate decarboxylase, oxalate‐degrading enzymes, hyperoxaluria, biological drug

## Abstract

Oxalate decarboxylase (OxDC) from *Bacillus subtilis* is a Mn‐dependent hexameric enzyme that converts oxalate to carbon dioxide and formate. OxDC has greatly attracted the interest of the scientific community, mainly due to its biotechnological and medical applications in particular for the treatment of hyperoxaluria, a group of pathologic conditions caused by oxalate accumulation. The enzyme has an acidic optimum pH, but most of its applications involve processes occurring at neutral pH. Nevertheless, a detailed biochemical characterization of the enzyme at neutral pH is lacking. Here, we compared the structural–functional properties at acidic and neutral pH of wild‐type OxDC and of a mutant form, called OxDC‐DSSN, bearing four amino acid substitutions in the lid (Ser161‐to‐Asp, Glu162‐to‐Ser, Asn163‐toSer, and Ser164‐to‐Asn) that improve the oxalate oxidase activity and almost abolish the decarboxylase activity. We found that both enzymatic forms do not undergo major structural changes as a function of pH, although OxDC‐DSSN displays an increased tendency to aggregation, which is counteracted by the presence of an active‐site ligand. Notably, OxDC and OxDC‐DSSN at pH 7.2 retain 7 and 15% activity, respectively, which is sufficient to degrade oxalate in a cellular model of primary hyperoxaluria type I, a rare inherited disease caused by excessive endogenous oxalate production. The significance of the data in the light of the possible use of OxDC as biological drug is discussed. © 2019 IUBMB Life, 1–11, 2019

**Abbreviations**
CHO‐GO: chinese hamster ovary cells stably expressing glycolate oxidase
CHO‐GO‐AGT: CHO‐GO cells stably expressing human AGT
NaGlyox: sodium glyoxylate
OxDC: oxalate decarboxylase
OxDC‐DSSN: OxDC bearing the Ser161Asp, Glu162Ser, Asn163Ser, Ser164Asn mutations
PH1: primary hyperoxaluria type 1
SEC: size exclusion chromatography

## INTRODUCTION

**Figure 1:**
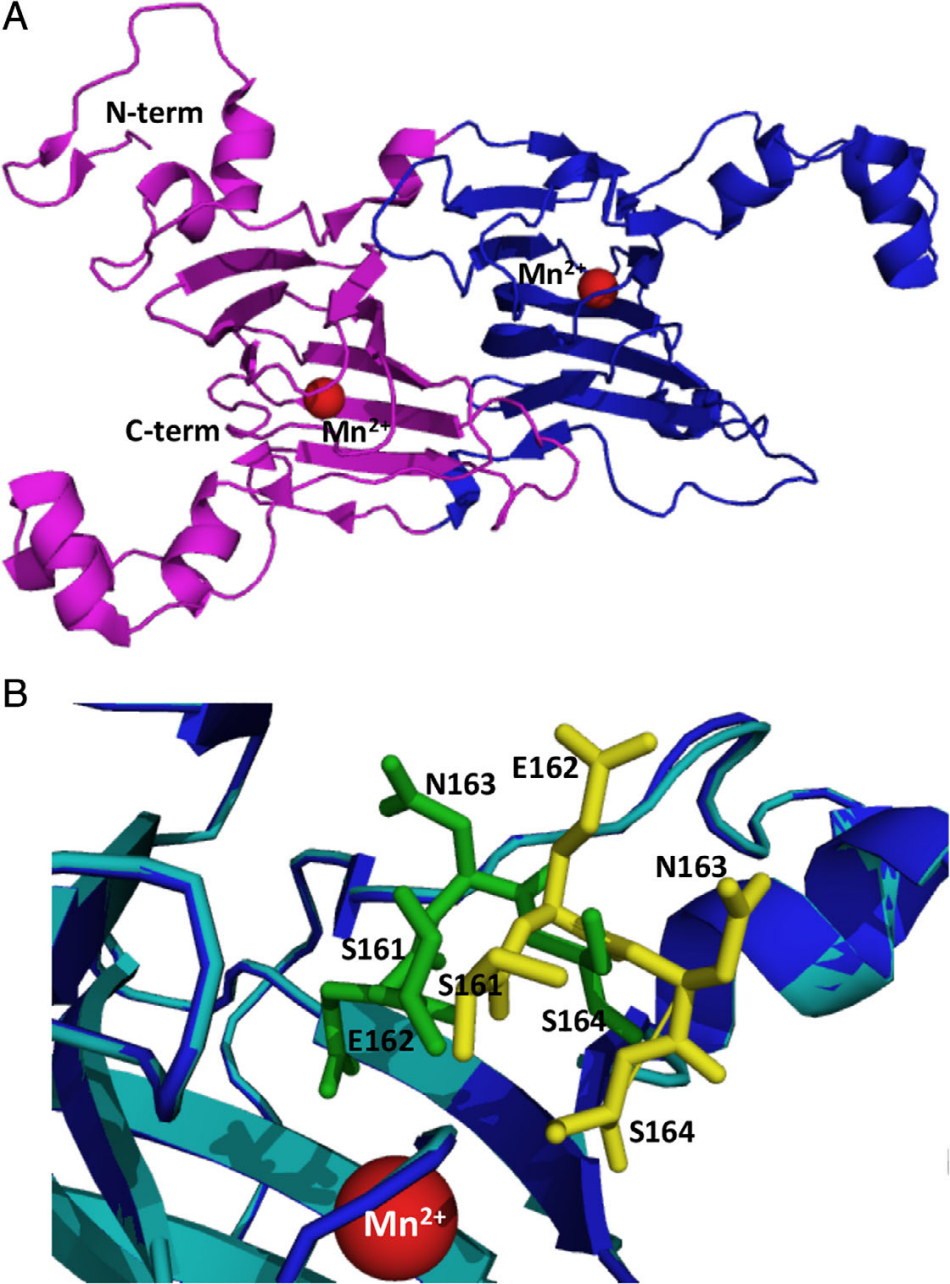
Structural representation of *B. subtilis* OxDC monomer and conformational orientation of the lid. (A) Structure of the OxDC monomer (PDB 1J58). Cupin domain I and cupin domain II are colored blue and purple, respectively. (B) Superimposition of the OxDC crystal structures in the closed (blue) and open (cyan) conformation (PDB 1J58 and 1UW8). The lid is represented by yellow and green sticks in the open and closed conformation, respectively. In both panels Mn(II) ion is represented as a red sphere. The figure was rendered using Pymol.

Oxalate decarboxylase (OxDC) from *Bacillus subtilis* requires Mn and O_2_ to catalyze the conversion of oxalate to formate and CO_2_ 1, 2. The enzyme is a homohexamer of 264 kDa made up of two trimeric units, and belongs to the cupin superfamily called bicupins. The monomer comprises two cupin domains (domain I residues 56–233; domain II residues 234–379 and 8–55), each formed by a characteristic β‐sandwich structure (Fig. 1A) 3, 4. A Mn(II) ion is located in the center of each cupin domain and has an octahedral geometry in which the metal interacts with highly conserved amino acids 4. Recently, it has been postulated that domain I is responsible for the decarboxylase activity, while domain II exclusively plays a structural role 5. This hypothesis is supported by numerous pieces of evidence, including the presence of a formate ion bound at the active site 3 and the identification of a suitable proton donor (Glu162) in domain I whose mutation completely abolishes catalytic activity 3, 4. Notably, crystallography studies have identified the presence in domain I of a channel for oxalate diffusion that can exist in an “open” or “closed” form as a result of the conformational rearrangement of a lid structure formed by residues 161–165 (Fig. 1B) 4. The open‐closed conformational change seems to be important to (i) allow access of the substrate to the active site, (ii) move Glu162 in an optimal position to protonate the formyl radical intermediate, and (iii) release the products 5.

In addition to the decarboxylase reaction, OxDC is also able to catalyze the oxygen‐dependent oxidation of oxalate to carbon dioxide generating hydrogen peroxide. The turnover number for the oxidase activity is 0.2% that of the decarboxylase activity 6. However, four amino acids substitutions in the OxDC lid (Ser161‐to‐Asp, Glu162‐to‐Ser, Asn163‐toSer, and Ser164‐to‐Asn) of the catalytic domain give rise to a variant, named OxDC‐DSSN, characterized by undetectable decarboxylase activity and a 116‐fold increased oxidase activity as compared with OxDC 7. The crystal structure of OxDC‐DSSN is not remarkably different with respect to the wild type, except for the conformation of the lid, which is intermediate between the open and the closed form 7.

OxDC has greatly attracted the interest of the scientific community, mainly due to its application in biotechnology. Fungal OxDC is employed to prevent the formation of oxalate salt deposits in industrial processes such as papermaking 8, 9 and beer production 10. At diagnostic level, OxDC is used for the determination of oxalic acid concentration in food and complex biological samples such as blood and urine 11, 12, 13. Finally, the enzyme has been proposed as a possible biological drug in the treatment of hyperoxaluria. Hyperoxaluria is a pathological condition in which oxalate, an end product of human metabolism normally excreted by urine, accumulates at high levels leading to calcium oxalate crystals precipitation in the urinary tract 14, 15 and to a potentially fatal condition named systemic oxalosis 16. The most severe form of disease is primary hyperoxaluria type I (PH1), a genetic disorder due to inherited mutations on the gene encoding alanine:glyoxylate aminotransferase (AGT). Recently, based on studies performed in mice 17, 18 and humans 19, it has been proposed that orally administered OxDC could degrade intestinal oxalate, thus reducing the absorption from exogenous sources and possibly promoting the clearance of the endogenous pool.

Although most of the therapeutic and industrial applications of OxDC depend on its activity under neutral conditions, the enzyme has an optimum pH around 4 and shows a remarkable drop in activity at increasing pH 20, 21, 22. Thus, efforts should be made to better define the properties of the enzyme at physiological pH, as the basis to improve its oxalate metabolizing activity under physiological conditions. However, a proper biochemical characterization of OxDC at neutral pH is lacking. In addition, as already mentioned, OxDC can be converted into an oxalate oxidase (OxDC‐DSSN) by site‐directed mutagenesis 7. Notably, some pieces of evidence allow to speculate that the oxalate oxidase reaction could be performed at neutral pH, possibly because the chemical groups involved in the catalytic mechanism maintain a proper protonation state and/or a proper orientation. In fact, some oxalate oxidases display an optimum pH around 7 23, 24, and a recent study reports that the encapsulation of oxalate oxidase from barley into polymeric zwitterionic capsules increases by twofold its activity at neutral pH 25. Since these enzymes derive from plant sources and their purification is not suitable for large‐scale applications, we thought to the possibility that the oxalate oxidase activity of OxDC‐DSSN could be exploited as an alternative option to degrade oxalate under physiological conditions. However, no information are available on the pH profile of this OxDC variant.

To fill these gaps, in this work we investigated the molecular properties of OxDC and OxDC‐DSSN at neutral pH. We found that both enzymes retain a low but detectable enzymatic activity at pH 7.2. However, at neutral pH, they display a lower thermal stability and, in the case of OxDC‐DSSN, a higher propensity to aggregation as compared with that at acidic pH. Notably, the residual activity of OxDC and OxDC‐DSSN at physiological pH is sufficient to detoxify oxalate in a cellular model of PH1. The possible implications of the results for the biotechnological applications of the enzyme are discussed.

## EXPERIMENTAL PROCEDURES

### Site‐Direct Mutagenesis

The cDNA of *B. subtilis* OxDC with a thrombine cleavage site and a 6‐Histidine (6xHis) tag at the C‐terminus cloned in a pET24a(+) vector (pET24a‐OxDC) was purchased from GeneScript (Piscataway, New Jersey, USA). The pET24a‐OxDC‐DSSN construct was obtained by introducing the Ser161Asp, Glu162Ser, Asn163Ser, and Ser164Asn mutations on the pET24a‐OxDC vector by site‐directed mutagenesis using the QuikChange II site‐directed mutagenesis kit (StratageneSan Diego, California). The oligonucleotides used for mutagenesis were 5′‐CTG CTG GTT TTT GAC GAT GGC AGC TTC AGC GAG AAC AGC ACC TTT CAA CTG ACC GAC TGG CTG GC‐3′ and its complement. Underlined codons indicate mutated amino acids. All the mutations were confirmed by the entire DNA sequencing.

### Protein Expression and Purification

His‐tagged OxDC and OxDC‐DSSN were expressed in *Escherichia coli* according to Reinhardt et al. 20 with minor modifications, and purified by affinity chromatography using HiTrap™ TALON® crude columns as described in Supporting Information (Supporting Information Fig. S1).

### Enzymatic Activity Measurement

Deacrboxylase and oxidase activities were measured according to the protocol previously described by Magro et al. 26 and Requena et al. 27, respectively, with minor modifications (see Supporting Information). The kinetic parameters of the decarboxylase and oxidase reactions were determined by measuring the enzymatic activity at potassium oxalate concentrations ranging from 0.1 to 250 mM and from 10 to 200 mM, respectively. Data were fitted to the Michaelis–Menten equation. For pH‐dependence studies, decarboxylase and oxidase activity assays were carried out using the buffers reported in Supporting Information Table S1. We performed the assays in the whole pH range at physiological ionic strength (154 mM) by keeping a constant NaCl concentration of 140 mM and modulating buffer concentration. Ranges of buffer overlaps were also included to ensure against specific buffer effects. In addition, in order to avoid changes of ionic strength and pH of the assay mixture, potassium oxalate was dissolved in the buffer used for each assay and the pH of each solution was checked. Finally, in order to verify the reliability of both the decarboxylase and the oxidase activity assay, formate and H_2_O_2_ standard curves were determined at each pH. In both cases, no significant pH‐dependent changes were noticed (Supporting Information Fig. S2).

### Spectroscopic Measurements

Absorption measurements were carried out using a Jasco V‐550 spectrophotometer with 1‐cm path length quartz cuvettes. The increase in turbidity following aggregation was monitored by measuring the absorbance at 600 nm as a function of time. Intrinsic fluorescence emission spectra were recorded on a Jasco FP‐750 spectrofluorometer equipped with a thermostatically controlled cell holder by using 1 cm path length quartz cuvettes. Excitation was set at 280 nm with both the excitation and the emission slits of 5 nm. CD spectra were registered by a Jasco‐710 spectropolarimeter. Thermal stability studies were performed by monitoring the CD signal at 222 nm of the enzymes upon increasing temperature from 25 to 90°C at a heating rate of 1.5 °C/min. Melting temperatures (*T*_m_) were obtained by fitting the data to a two‐state unfolding model using the Origin Pro7 software according to the method of Pace 28. Dynamic light scattering (DLS) measurements were performed on a Zetasizer Nano S device (Malvern Instruments, Malvern, UK) equipped with a Peltier temperature controller by using disposable 12.5 × 45‐mm cells with stopper. All spectroscopic measurements were carried out at 0.5 mg/mL enzyme concentration, 25°C, in 52 mM sodium acetate pH 4.2 containing 140 mM NaCl and/or in 16 mM Tris–HCl pH 7.2 containing 140 mM NaCl.

### Size‐Exclusion Chromatography and Cross‐Linking Studies

Size‐exclusion chromatography (SEC) analyses were performed on a Superdex 200 10/300 GL column (GE Healthcare, Chicago, IL) using an Akta FPLC system (GE Healthcare, Chicago, IL). The experiments were carried out at 0.5 mg/mL protein concentration in 16 mM Tris–HCl pH 7.2, 140 mM NaCl.

Cross‐linking of 9 μM OxDC or OxDC‐DSSN was performed in 52 mM sodium acetate pH 4.2 and 140 mM NaCl buffer with 1% glutaraldehyde for 2 min at 25°C. The reaction was quenched in 1 M Tris–HCl pH 8 and 7 μg of protein were then analyzed by SDS‐PAGE.

### Cell Culture and Lysis

Chinese Hamster Ovary cells stably expressing glycolate oxidase (CHO‐GO) were cultured at 37°C under O_2_/CO_2_ (19:1) in Ham's F12 Glutamax medium (Thermo‐Fisher, Carlsbad, CA) supplemented with fetal bovine serum (10%, v/v), penicillin (100 units/mL), and streptomycin (100 μg/mL). CHO‐GO cells stably expressing human AGT (CHO‐GO‐AGT) were cultured in the presence of 1 mg/mL geneticin (G418). Recombinant purified OxDC and OxDC‐DSSN, in the untagged or in the His‐tagged form, were transduced into the cells using the Xfect™ Protein Transfection kit (Clontech Laboratories, Mountain View, CA) according to the manufacturer's instructions. After 24‐h incubation, cells were harvested and lysed in phosphate‐buffered saline as previously described 29. Protein concentration was determined using the Bradford assay.

### Glycolate toxicity assay

CHO‐GO and CHO‐GO‐AGT cells were seeded into 96‐well plates at a density of 8,000 cells/well. After 24 h, CHO‐GO cells were subjected to protein transfection with OxDC or OxDC‐DSSN as explained above. Upon further 24‐h incubation, CHO‐GO, CHO‐GO‐OxDC, CHO‐GO‐OxDC‐DSSN, and CHO‐GO‐AGT cells were treated with Hepes buffered glycolate at pH 7.0 to a final concentration of 1 mM, to induce glyoxylate production, or with Hepes buffer alone, as untreated negative control. Cell viability was evaluated after 24‐h incubation by crystal violet staining as previously reported 30. The percentage of viability was calculated from the ratio between the absorbance of the sample treated with glycolate and that of the corresponding untreated control.

### Oxalate Determination

CHO‐GO and CHO‐GO‐AGT cells were seeded into 24‐well plates at a density of 8,000 cells/well. After 24 h, CHO‐GO cells were subjected to protein transfection with purified OxDC or OxDC‐DSSN as explained above. Upon further 24‐h incubation, CHO‐GO, CHO‐GO‐OxDC, CHO‐GO‐OxDC‐DSSN, and CHO‐GO‐AGT cells were treated with Hepes buffered glycolate at pH 7.0 to a final concentration of 1 mM, to induce oxalate production. After 24‐h incubation, the medium was collected, incubated with activated charcoal, and the oxalate levels were determined by using a commercial kit (Trinity Biotech, Bray, Ireland) accordingly to the protocol of Miyata et al 31. In order to exclude any interference of the medium, we determined a calibration curve by dissolving known amounts of oxalate in culture medium, together with a blank sample.

### Western Blot

A volume of 20 μg of cell lysate or 0.1 μg of purified proteins were loaded per lane on a 10 or 7.5% polyacrylamide gel along with the Precision plus protein Kaleidoscope™ (Bio‐Rad, hercules, CA) molecular mass markers. Following transfer on a nitrocellulose membrane by the iBlot device (Thermo‐Fisher, Carlsbad, CA) the membrane was blocked in 5% bovine serum albumin (BSA) for 1 h at 37°C. For OxDC‐His and OxDC‐DSSN‐His detection, the membrane was incubated with an anti‐His (C‐Term)‐HRPs conjugate antibody (Thermo‐Fisher R931‐25, dilution 1:5000) for 1 h at RT. GAPDH (6‐C5; 1:500, Santa Cruz Biotechnologies, Dallas, TX) antibody was used as loading control. Blotted proteins were detected with ECL® (Thermo‐Fisher, Carlsbad, CA), using a ChemiDoc XRS Imaging System (Bio‐Rad, Hercules, CA).

### Statistical Analysis

Experiments have been performed at least in triplicate and in any case the standard error mean was less than 10%. Statistical analysis was performed by using Origin® 7.03 (Origin Lab) or GraphPad Prism Version 5.0 (GraphPad software, San Diego, CA, USA).

## RESULTS AND DISCUSSION

### Kinetic and Structural Properties of OxDC and OxDC‐DSSN

By a two‐step purification process, we obtained untagged OxDC and OxDC‐DSSN with a yield of 20 mg of pure protein per liter of bacterial culture (Supporting Information Fig. S2). At pH 4.2 in the presence of *o*‐phenylenediamine (*o*‐PDA), purified OxDC displays a OxDC‐specific activity of 83 U/mg in line with previous reports 6, thus suggesting that our purification strategy did not alter protein functionality and/or Mn binding. Although *o*‐PDA is a known OxDC activator 32, it is highly toxic (*o‐*PDA CAS No. 95‐54‐5). Thus, we also determined OxDC activity at pH 4.2 in the absence of *o*‐PDA in the assay mixture and we found that OxDC displayed a significantly lower decarboxylase specific activity (28 U/mg) and a K_m_ for oxalate of 8 mM. As expected, the mutations of lid residues Ser161Asp, Glu162Ser, Asn163Ser, Ser164Asn in OxDC‐DSSN completely abolished the decarboxylase activity, but gave rise to an enzyme displaying a detectable oxalate oxidase activity, in the same range of that already reported (Table 1) 7.

**Table 1:** Kinetic parameters for the oxalate decarboxylase and oxidase activity of OxDC and OxDC‐DSSN, respectively, at pH 4.2 and 7.2

Decarboxylase activity				Oxidase activity			
	*V*_max_ (U mg^−1^)	K_m_ (mM)	k_cat_ (s^−1^)		*V*_max_ (U mg^−1^)	K_m_ (mM)	k _cat_ (s^−1^)
OxDC pH 4.2	28.0 ± 2.0	8.0 ± 0.8	20.0 ± 1.8	OxDC‐DSSN pH 4.2	2.0 ± 0.1	0.30 ± 0.07	1.4 ± 0.2
OxDC pH 7.2	2.0 ± 0.4	2.0 ± 1.0	1.45 ± 0.15	OxDC‐DSSN pH 7.2	0.35 ± 0.04	70.0 ± 20.0	0.85 ± 0.07

**Figure 2:**
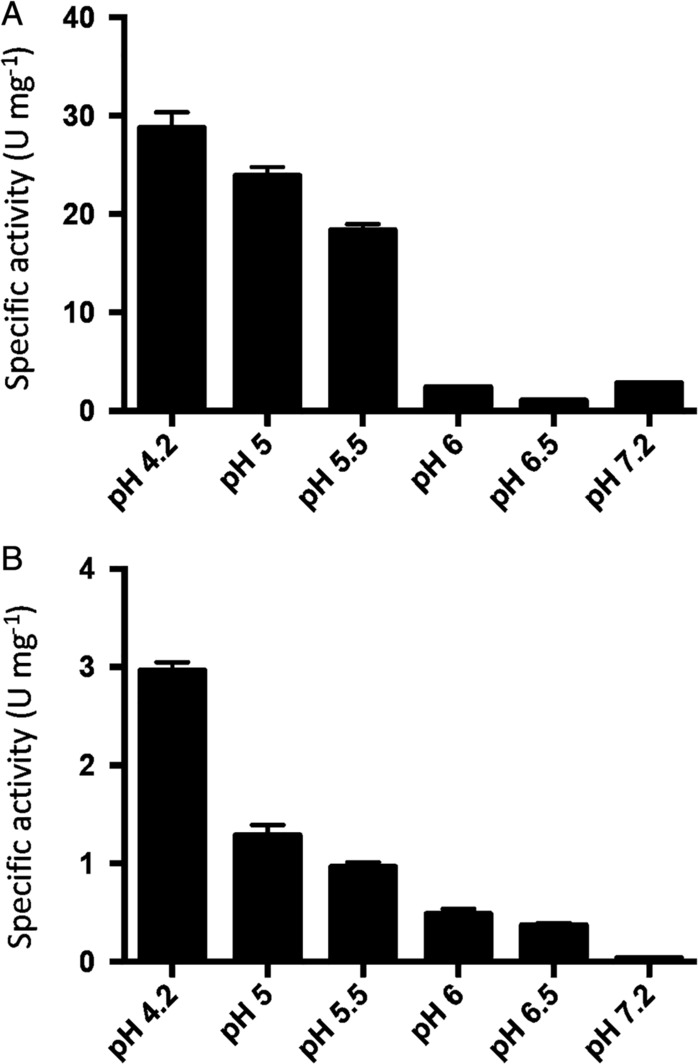
Effect of pH on OxDC and OxDC‐DSSN activity. Histogram representing specific activity values of OxDC decarboxylase reaction (A) and OxDC‐DSSN oxidase reaction (B) at different pHs. Protein concentration was 10 μg/mL. Data are representative of two independent experiments. Bar graphs represent the mean ± SEM. The buffers used and the technical details are reported in Supporting Information.

We measured the decarboxylase and oxidase activity of OxDC and OxDC‐DSSN, respectively, in the pH range 4.2–7.2. The upper limit of the analysis was chosen taking into account that we aimed at testing the functionality of the two enzymes in a cellular model of PH1 (see below) and that the pH value of the cell cytosol is 7.2 33. Appropriate controls were performed to validate each assay in the whole pH range, as well as to evaluate possible buffer effects. Details are given in Materials and Methods and Supporting Information sections. We found that the activity of both enzymes significantly decreases at increasing pH (Fig. 2), in line with previous reports 20, 21. However, at pH 7.2 the two proteins retain 7 and 15% activity, respectively. It should be observed that OxDC activity is higher at pH 7.2 than at pH 6.5, showing specific activity values of 2.0 ± 0.3 U mg^−1^ and 1.1 ± 0.1 U mg^−1^, respectively. Considering that (i) assay calibration curves obtained at various pH values allow to exclude any influence of the coupled assay, (ii) in the pH range 6–7.2, the values are near to the limit of quantification of the assay, but the coefficient of inter‐assay variation is below 15%, we cannot exclude that the observed differences could have a biological meaning possibly related to the biochemical mechanism of action of OxDC in the neutral range. Unfortunately, we could not increase the signal by increasing protein concentration or incubation time, because of the strong tendency of the protein to aggregation (see below).

Nevertheless, we determined the kinetic parameters for the decarboxylation or oxidation reaction at pH 7.2. As shown in Table 1, OxDC shows a 13‐fold reduction in the *V*_max_ of the decarboxylation reaction, without significant alterations of the K_m_ value for oxalate as compared to pH 4.2. On the other hand, OxDC‐DSSN shows a 7‐fold reduced *V*_max_ for the oxidation reaction and a remarkably increased K_m_ for oxalate as compared to pH 4.2. These findings are in contrast with previous results 20, 21, 34, 35, which reported that OxDC activity is undetectable at neutral pH due to the fact that only monoprotonated oxalate is a productive substrate. However, an in‐depth analysis of the OxDC kinetic properties at neutral pH has never been performed. Moreover, a recent report suggests that the pH‐dependence of catalytic activity could be also governed by small changes in the active site of the protein that could stabilize the +3 oxidation state of the Mn ion 21. In addition, Moomaw et al. 36 reported that the change in oxalate oxidase activity as a function of pH is influenced by the protonation state of an active site Asp residue at position 241. All these considerations could possibly explain why the measured activity of OxDC and OxDC‐DSSN at pH 7.2 does not correspond to that expected based on the amount of monoprotonated oxalate present in solution. In this regard, pieces of recent experimental evidence indicate that (i) OxDC activity is detectable in *E. coli* cells grown at pH 7 37, (ii) an oral formulation of recombinant OxDC in its crystalline form shows 2% residual activity under physiological conditions and is able to metabolize intestinal oxalate in a mouse model of hyperoxaluria 17, and (iii) OxDC is able to degrade oxalate contained in spinach leaves under conditions mimicking the intestinal environment 38. Finally, Albert et al. recently demonstrated that HEK293 cells transfected with a construct encoding OxDC (HEK293‐OxDC) exhibit a higher viability as compared to untransfected HEK293 cells when exposed to oxalate stress. Moreover, HEK293‐OxDC, but not HEK293 cells, are able to metabolize oxalate added to the medium, thus suggesting that OxDC is biologically active in the cytoplasm 39. These results provide indirect evidence for the presence of residual enzymatic activity at neutral pH. It should be mentioned that the oxidase‐specific activity of OxDC‐DSSN is lower than the decarboxylase specific activity of OxDC. Nevertheless, the finding that some oxalate oxidases active at neutral pH have been identified 23, 24, 25 supports future studies of rational and/or irrational design of this construct.

**Figure 3:**
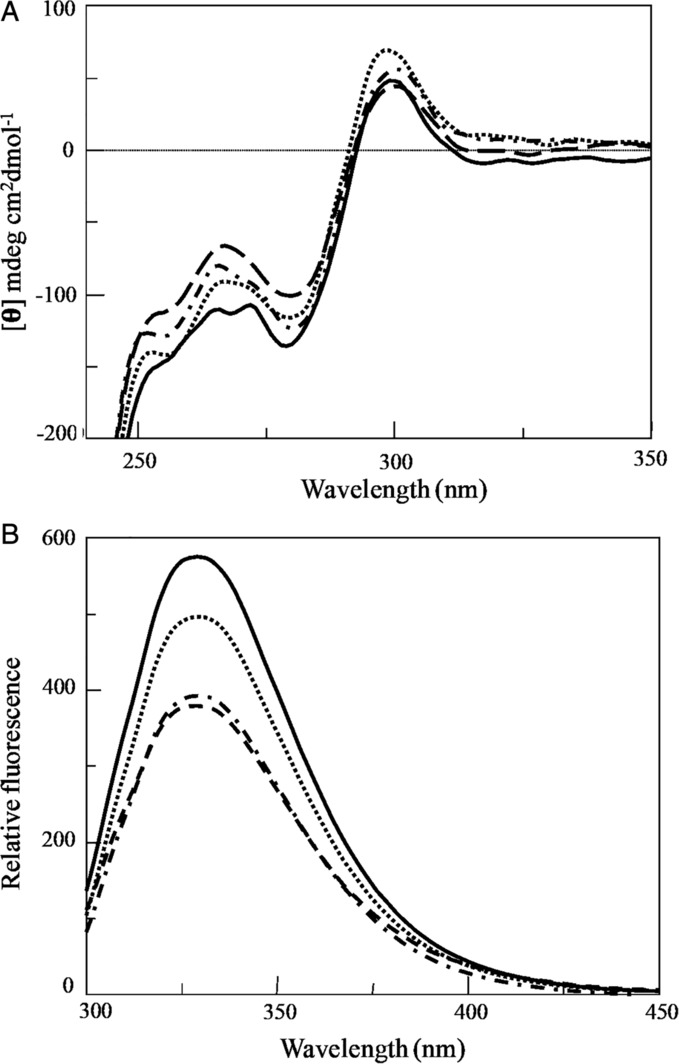
Spectroscopic features of OxDC and OxDC‐DSSN (A) Near‐UV CD spectra and (B) intrinsic fluorescence emission spectra (λ_exc_ 280 nm) of OxDC at pH 4.2 (—) or pH 7.2 (• •) and of OxDC‐DSSN at pH 4.2 (‐‐‐) or pH 7.2 (‐ • ‐). Spectra were registered at 0.5 mg/mL enzyme concentration in 52 mM sodium acetate pH 4.2, 140 mM NaCl or 16 mM Tris–HCl pH 7.2, 140 mM NaCl.

To investigate if any structural change could occur in OxDC and OxDC‐DSSN at physiological pH, we compared their spectroscopic properties at acidic and neutral pH maintaining a physiological ionic strength. At pH 4.2, the two proteins exhibited almost identical far‐UV (data not shown) and near‐UV CD spectra (Fig. 3A). No significant alterations were noticed at pH 7.2. Upon excitation at 280 nm (Fig. 3B), both OxDC and OxDC‐DSSN exhibited an intrinsic fluorescence emission spectrum with maximum at 329 nm, indicating a folded structure. However, the emission intensity of OxDC‐DSSN was about 1.5‐fold lower than that of OxDC, probably as a consequence of the fact that the mutation of lid residues could affect the orientation of some aromatic residues.

**Figure 4:**
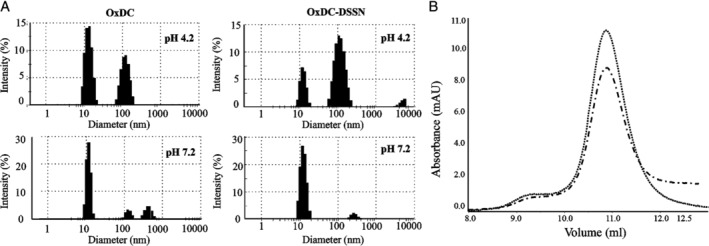
Analysis of the OxDC and OxDC‐DSSN quaternary structure. (A) DLS analysis of the size distribution of 0.5 mg/mL OxDC and OxDC‐DSSN at pH 4.2 (52 mM sodium acetate pH 4.2, 140 mM NaCl) and pH 7.2 (16 mM Tris–HCl pH 7.2, 140 mM NaCl). (B) Elution profile of OxDC (…) and OxDC‐DSSN (.‐.‐) at 0.5 mg/mL concentration loaded on a Superdex 200 10/300 GL column (GE Healthcare) equilibrated and run in 16 mM Tris–HCl pH 7.2 containing 140 mM NaCl. Detection was set at 280 nm.

DLS analyses revealed that at both acidic and neutral pH OxDC and OxDC‐DSSN display a peak with a diameter of 12 ± 1 nm, corresponding to an hexamer, and a second peak at approximately 200 nm, corresponding to aggregates (Fig. 4A). Considering that the scattering intensity is proportional to the sixth power of a particle diameter 40, the hexamer represents by far the most abundant species in solution, thus suggesting that neither the mutation of residues of the lid nor the change of the pH affects the quaternary structure of the enzyme. We confirmed these data by SEC experiments, which were only performed at pH 7.2 due to the technical limitations of the Superdex 200 10/300 GL column. In line with previous data, OxDC eluted from the column with a main peak at 10.8 mL, corresponding to the elution volume of an hexamer, along with a shoulder at 9.3 mL, corresponding to an aggregated form 35. We found a similar elution profile for OxDC‐DSSN, although the area of the hexamer is 1.5‐fold lower with respect to that of OxDC (Fig. 4B). Since no other peaks were present in the elution profile of OxDC‐DSSN, and considering that the two proteins have a similar molar extinction coefficient at 280 nm, the reduced peak area is probably a consequence of the formation of high‐molecular weight aggregates that do not enter the column bed (see below), as previously reported for other proteins 41, 42. The technical features of the SEC column do not allow to carry out runs at pH 4.2. Therefore, we could only performed cross‐linking experiments that confirmed the presence of the hexamer for both proteins, along with tetrameric, dimeric, and monomeric species deriving from the limited efficiency of the cross‐linking reactions (Supporting Information Fig. S3).

Overall, spectroscopic data indicate that OxDC and OxDC‐DSSN do not undergo gross changes of their secondary, tertiary, and quaternary structure at neutral pH with respect to acidic pH. Although this is not surprising considering that the available crystal structures have been solved at neutral or alkaline pH 3, 4, the results confirm that the reduced catalytic activity observed at increasing pH does not depend on a significant conformational change of the protein. Rather, it is merely a catalytic problem related to the protonation state of the substrate and/or of active site groups as discussed above 2, 20, 36.

### Stability and Aggregation Propensity of OxDC and OxDC‐DSSN at pH 4.2 and 7.2

**Figure 5:**
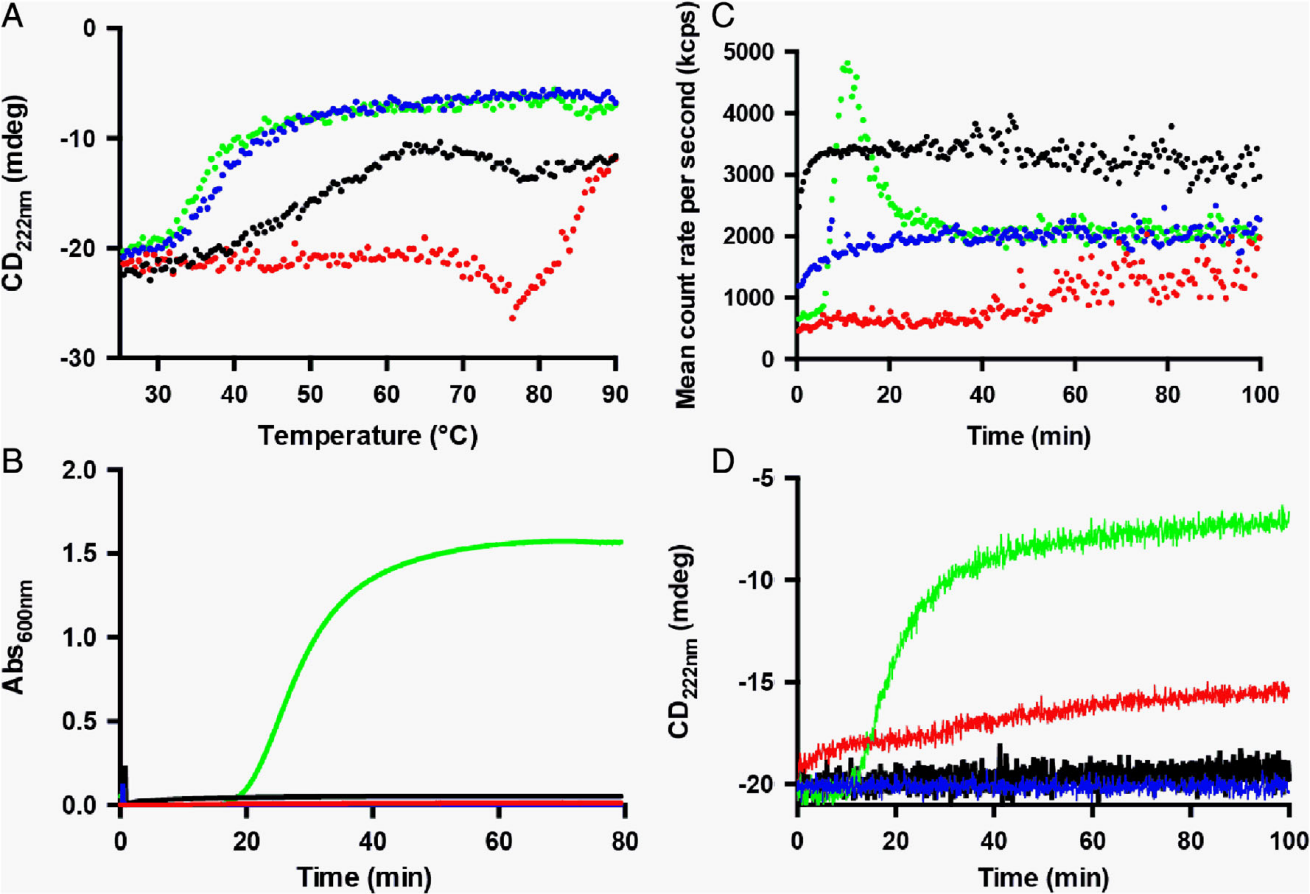
Thermal stability and aggregation propensity of OxDC and OxDC‐DSSN at pH 4.2 and 7.2. (A) Change in the CD signal at 222 nm at increasing temperature from 25 to 90°C (heating rate 1.5 °C/min). (B) Absorbance changes at 600 nm as a function of time, (C) change in the total count rate (measured as kilo counts per second) and (D) CD signal at 222 nm as a function of time. All measurements were performed at 0.5 mg/mL enzyme concentration, in 16 mM Tris–HCl pH 7.2, 140 mM NaCl or 52 mM sodium acetate pH 4.2, 140 mM NaCl, at 25°C. The color code is the following: red, OxDC pH 4.2; blue, OxDC pH 7.2; black, OxDC‐DSSN pH 4.2; green, OxDC‐DSSN pH 7.2.

Considering the possible use of OxDC and/or OxDC‐DSSN as biological drugs, we compared their thermal stability at pH 4.2 and 7.2 and physiological ionic strength by monitoring the decrease of dichroic signal at 222 nm indicative of the secondary structure content. At pH 4.2 OxDC maintains its secondary structure up to 60°C and shows a transition with mid‐point at 69°C to a conformation characterized by a higher signal, followed by a second transition with mid‐point at 85°C leading to the almost complete loss of secondary structure content (Fig. 5A). This behavior, which is frequently observed during acid protein unfolding 43, suggests a two‐step process consisting in the first formation of a partly folded intermediate characterized by a pre‐molten globule‐like structure and its subsequent conversion to the fully unfolded state. On the other hand, OxDC‐DSSN undergoes a progressive loss of secondary structure from 36 to 64°C, with a mid‐point around 47°C, followed by an apparent recovery of secondary structure with mid‐point at 85°C and by the subsequent almost complete loss of the dichroic signal (Fig. 5A). At pH 7.2, both OxDC and OxDC‐DSSN show a classical two‐state unfolding process with mid‐point transitions at 38 and 36°C, respectively.

Thus, although OxDC and OxDC‐DSSN do not seem to undergo major structural changes at neutral pH, they are less resistant to thermal stress with respect to acidic pH. This finding is not easy to explain at structural level, but it can be speculated that the protonation state of key protein residues, whose identity is presently unknown, could be crucial for the maintenance of the native structure although it does not affect the overall conformation of the protein. In addition, it is worth nothing that OxDC‐DSSN displays a lower resistance with respect to OxDC, thus suggesting that one or more of the mutations altering the reaction specificity could also induce some local conformational change that could promote unfolding.

Similar conclusions were also drawn by looking to the aggregation propensity of OxDC and OxDC‐DSSN at pH 4.2 and 7.2, physiological ionic strength. Turbidimetry experiments (Fig. 5B) did not reveal aggregation of both enzymes up to 60 min at pH 4.2, as well as for OxDC at pH 7.2. Accordingly, we did not notice the formation of significant amounts of aggregates in DLS analyses, as shown by the presence of the signal corresponding to the dimer throughout the analysis (Supporting Information Fig. S4). On the other hand, for OxDC‐DSSN at pH 7.2 we detected a time‐dependent increase in the absorbance at 600 nm, indicative of an ongoing aggregation process. By fitting the signal changes at 600 nm vs time we estimated a *t*_1/2_ of aggregation equal to 28.4 ± 0.3 min. Under the same experimental conditions, a rapid increase in DLS count rate occurred after a 6 min lag phase, followed by a decrease of the signal probably due to the precipitation of insoluble high molecular weight aggregates (Fig. 5C). Accordingly, the signal of the OxDC‐DSSN hexamer disappeared after 9 min and high molecular weight aggregates (200–1,200 nm) appeared (Supporting Information Fig. S4). Finally, the increase of the count rate in DLS is paralleled by a progressive decrease of the CD signal at 222 nm, thus suggesting that aggregation is associated with an unfolding process (Fig. 5D).

**Figure 6:**
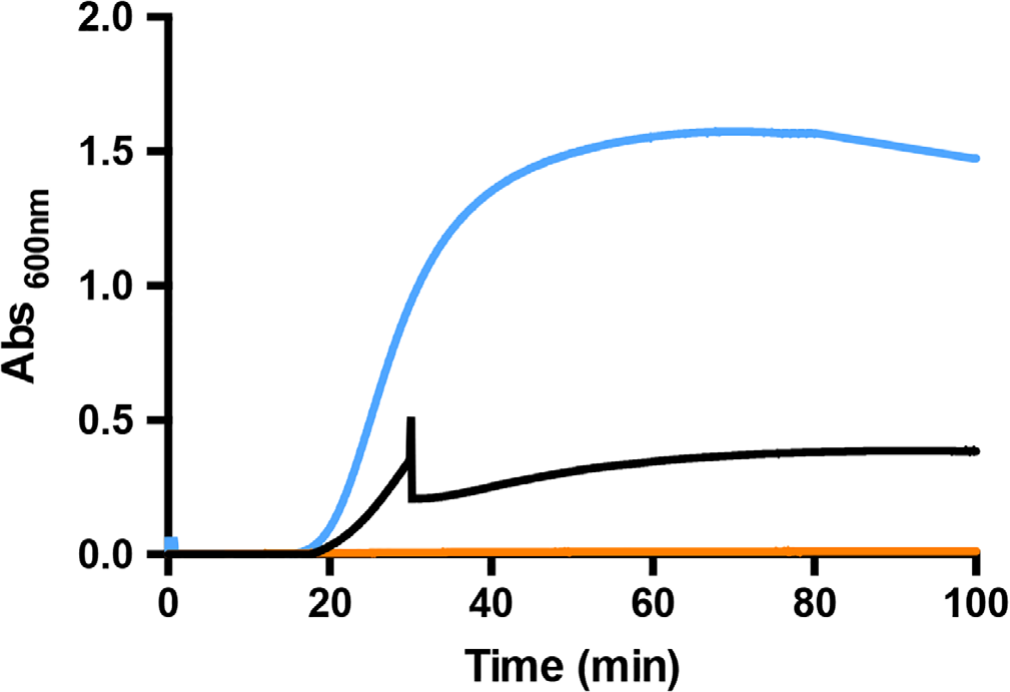
Time‐dependent aggregation of OxDC‐DSSN at pH 7.2 in absence or presence of sodium glyoxylate. Absorbance changes at 600 nm as a function of time of OxDC‐DSSN at 0.5 mg/mL concentration in the absence (light blue) or presence (orange) of 50 mM sodium glyoxylate. The black line shows the absorbance changes occurring upon addition of sodium glyoxylate during aggregation (at the time indicated by the arrow). The buffer was 16 mM Tris–HCl pH 7.2, 140 mM NaCl, at 25°C.

Overall, these results agree with thermal stability studies and confirm an enhanced propensity of OxDC‐DSSN to unfold and aggregate at neutral pH with respect to OxDC. Although crystallographic analyses did not reveal major structural differences between the two proteins 7, in OxDC‐DSSN the lid remains in an intermediate form between the open and closed position, thus causing the exposure of residues located in the channel connecting the protein surface with the active site 7. Based on these considerations, we hypothesized that the use of a substrate analogue, such as sodium glyoxylate (NaGlyox), could induce at least a partial closure of the active site channel and consequently prevent aggregation. Thus, in order to better understand the mechanism leading to OxDC‐DSSN aggregation, we monitored the change in turbidity at pH 7.2 and physiological ionic strength upon addition of OxDC‐DSSN to the buffer in the absence or presence of 50 mM NaGlyox (Fig. 6). We found that NaGlyox prevents aggregation and, if added to the mixture during aggregation, it is able to stop the process, as shown by the progressive stabilization of the 600 nm absorbance signal to a value lower than that observed in the absence of ligand. The latter effect cannot be ascribed to dilution, since the addition of NaGlyox led to a less than 20% change in the overall volume of the mixture. Thus, we can conclude that the ligand at the active site is able to partly rescue for the conformational changes induced by amino acid substitutions in OxDC‐DSSN. It should be mentioned that the addition of NaGlyox is not suitable for the biotechnological application of the enzyme, since it would affect the enzymatic activity. Nevertheless, these findings confirm the hypothesis that OxDC‐DSSN aggregation comes from the increased flexibility of the lid and could be instrumental for a possible future engineering to improve the stability of the protein.

### Oxalate Detoxification Ability of OxDC and OxDC‐DSSN in a PH1 Cellular Model

**Figure 7:**
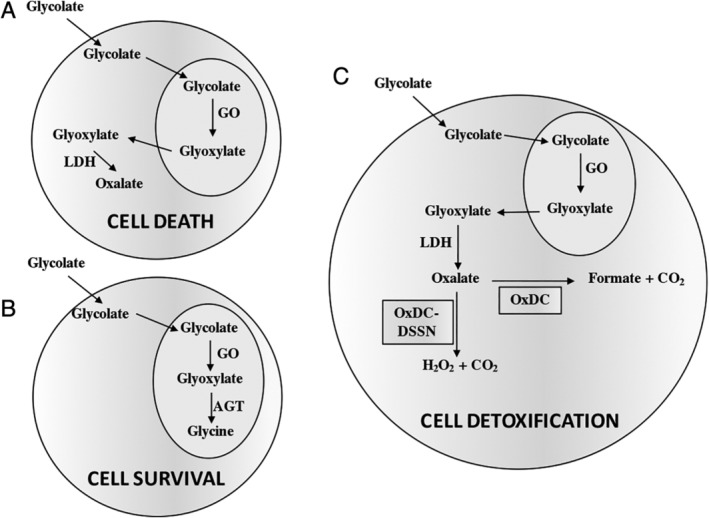
Schematic representation of oxalate detoxification in CHO‐GO cells. (A) Negative control: CHO‐GO cells (B) Positive control: CHO‐GO‐AGT cells; (C) CHO‐GO cells after OxDC or OxDC‐DSSN transfection. GO, glycolate oxidase; LDH, lactate dehydrogenase AGT, alanine:glyoxylate aminotransferase; CO_2_, carbon dioxide; H_2_O_2_, hydrogen peroxide.

**Figure 8:**
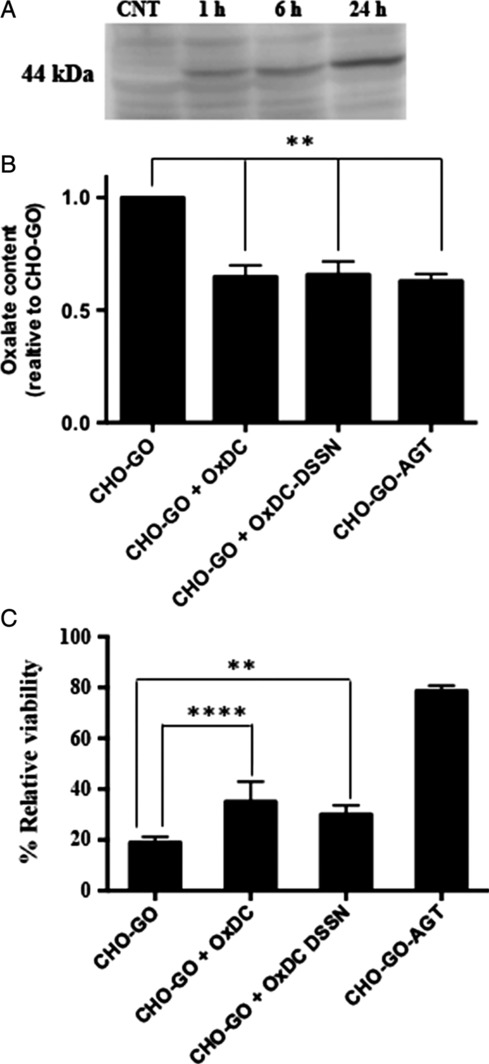
Oxalate detoxification in a cellular system. (A) CHO‐GO cells were transfected with OxDC‐His. At different times, cells were harvested and lysed. 20 μg of lysate were subjected to SDS‐PAGE and immunoblotted with anti‐His (C‐term) antibody (1:5000). GAPDH was used as loading control. Lanes are coded as follows: CNT: non‐transfected CHO‐GO cells; 1 h: cells harvested 1 h after transfection; 6 h: cells harvested 6 h after transfection; 24 h: cells harvested 24 h after transfection. (B) Amount of oxalate present in the culture medium of the indicated cell clones upon 24‐h treatment with 1 mM glycolate, expressed as relative oxalate content with respect to the negative control. Three replicates have been measured for each sample. Bars represent mean ± SEM. * *P* < 0.05. (C) Indirect glycolate toxicity assay. Histogram representative of cell viability after 24 h of treatment with 1 mM glycolate expressed as percentage with respect to untreated control. Six replicates have been measured for each sample. Bars represent mean ± SEM; ** *P* < 0.01, **** *P* < 0.0001.

Considering the potential use of an oxalate‐degrading enzyme for the treatment of hyperoxaluria, we wonder if at neutral pH the residual decarboxylase or oxidase activity of OxDC and OxDC‐DSSN, respectively, endowed the two proteins with the ability of metabolizing oxalate in a cellular environment. To test this hypothesis, we employed a PH1 cellular model made of CHO‐GO cells 44. In the presence of glycolate in the culture medium, CHO‐GO cells generate glyoxylate inside peroxisomes thanks to the presence of glycolate oxidase. Glyoxylate accumulates and is oxidized to oxalate in the cell cytosol, thus promoting cell death (Fig. 7). We transfected recombinant purified OxDC or OxDC‐DSSN in CHO‐GO cells by using the Xfect Protein Transfection Reagent. A preliminary experiment using OxDC‐His (Fig. 8A) revealed that the protein is detectable inside the cell starting from 1 h incubation and reaches its maximum level after 24 h. Thus, we evaluated the ability of CHO‐GO‐OxDC cells and CHO‐GO‐OxDC‐DSSN cells to metabolize oxalate and to survive in the presence of glycolate 24 h after protein transfection. As negative control, we used non‐transfected CHO‐GO cells, which lack any detoxifying enzyme. As positive control, we used CHO‐GO cells stably expressing AGT, which prevents oxalate formation by catalyzing glyoxylate conversion to glycine (Fig. 7). As shown in Fig. 8B, as compared with that of CHO‐GO, the culture medium of CHO‐GO‐OxDC and CHO‐GO‐OxDC‐DSSN cells treated with glycolate contains a significantly lower amount of oxalate, in line with that present in the medium of CHO‐GO‐AGT cells. This would imply that transfected cells are able to detoxify oxalate produced inside the cell. In particular, the residual activity at pH 7.2 of OxDC and OxDC‐DSSN encapsulated inside CHO‐GO cells (equal to 7 and 15%, respectively, as compared with the value at pH 4.2) was sufficient to reduce the concentration of oxalate released in the culture medium from 678 to 298 and 291 μM, respectively, over 24‐h incubation at 37°C. To confirm these data, we determined the viability of each cell clone upon treatment with glycolate. Although we verified that the protein transfection process did not affect cell viability *per se*, in order to exclude any possible bias, for each cell clone we obtained the viability value from the ratio between each glycolate‐treated sample and its untreated control. As expected, after treatment with glycolate CHO‐GO cells and CHO‐GO‐AGT cells showed a viability of 18 and 80%, respectively (Fig. 8B) 29. Interestingly, CHO‐GO‐OxDC cells and CHO‐GO‐OxDC‐DSSN cells showed a significant increase in viability of about 1.8‐fold and 1.6‐fold, respectively, as compared with CHO‐GO cells. Overall, these data indicate that both enzymes, although with a different efficiency, are able to degrade oxalate formed in the cell cytosol, thus suggesting that they could be able to metabolize oxalate under conditions similar to those present in biological fluids. These results are in line with those recently obtained in HEK293 cells 39.

## CONCLUSIONS

The use of OxDC for industrial and therapeutic applications has greatly attracted the interest of the scientific community in the last years. One of the main concerns on the use of this enzyme is related to its extremely low optimum pH of activity. In an attempt to explore the potential of OxDC as biological drug, we deeply investigated its biochemical properties at neutral pH. Our main findings indicate that wild‐type OxDC and a mutated form showing oxalate oxidase activity (OxDC‐DSSN) (i) retain detectable decarboxylase or oxidase activity, respectively, at neutral pH, (ii) do not undergo gross structural changes at neutral with respect to acidic pH, except for a reduced thermal stability associated in OxDC‐DSSN to an enhanced propensity to unfolding and aggregation, and (iii) are able to degrade oxalate endogenously formed in a cellular model of PH1.

These data provide the proof of principle for the possible use of oxalate degrading enzymes as biological drugs for the treatment of primary and enteric hyperoxalurias, pathologic conditions caused by the accumulation of oxalate from endogenous or exogenous sources, respectively. Up to now, most of the efforts have been directed to decrease intestinal oxalate absorption by oral formulations of OxDC named Nephure™, OxDC CLEC, and Oxazyme. The first is OxDC from *Synechococcus elongatus* used as a food ingredient 45, while OxDC CLEC and Oxazyme are forms of OxDC from *B. subtilis* currently under clinical development, proven to be effective in healthy subjects 17, 18, 19. These treatments are directed to secondary hyperoxaluria, because in the case of primary hyperoxalurias accumulated oxalate comes from endogenous sources. Our results, by unraveling the ability of OxDC and OxDC‐DSSN to metabolize oxalate inside the cell, suggest their possible development as biological drugs to treat primary hyperoxalurias. Nevertheless, it should be mentioned that although OxDC and OxDC‐DSSN retain a detectable specific activity at neutral pH, their stability is decreased, even if to a different extent. Since the aggregation propensity represents a big issue for a biological drug, protein engineering studies will be necessary to obtain a more stable enzyme. In addition, the direct administration of the enzyme is not feasible, because it would generate a remarkable immune response on the patients. However, as already done in other enzymatic deficits such as phenylketonuria 46, 47 appropriate delivery systems can be designed to profit from the metabolizing activity while being protected from the interaction with the immune system.

## CONFLICT OF INTEREST

The authors declare that there is no conflict of interest in the publication of this article.

## AUTHOR CONTRIBUTIONS

BC conceived the project; BC and EO designed experiments; CC, EO, and MD performed experiments; BC, CC, EO, LR, and MM analyzed and discussed the data; BC and EO wrote the manuscript.

## Supporting information


**Appendix S1**: Supporting Information
