# Supplementary material for: Biochemical properties and oxalate‐degrading activity of oxalate decarboxylase from bacillus subtilis at neutral pH
Source: IUBMB Life. 2019 Feb 26;71(7):917–27. doi: 10.1002/iub.2027 (PMC6850040; doi:10.1002/iub.2027)
Supplement: Supplementary file 1 — Appendix S1: Supporting Information [file IUB-71-917-s001.docx]

**Biochemical properties and oxalate-degrading activity of oxalate decarboxylase from *Bacillus subtilis* at neutral pH**

**Carolina Conter^1†^, Elisa Oppici^1†*^, Mirco Dindo^2^, Luigia Rossi^3^, Mauro Magnani^3^ and Barbara Cellini^2^**

**Supporting Information**

***OxDC and OxDC DSSN expression and purification***

*E. coli* BL21 (DE3) Codon plus cells transformed with the constructs pET24a-OxDC or pET24a-OxDC-DSSN were grown in Luria Broth (LB) in a total volume of 4.5 litres at 37 °C. Cells were grown with vigorous shaking to an OD of 0.3-0.4 at 600 nm and then heat-shocked at 42°C for 20 minutes. 5 mM MnCl_2_ was added to the culture and protein expression was induced with 0.5 mM IPTG for 16 hours at 30°C. Cells were then harvested by centrifugation and resuspended in lysis buffer (50 mM Tris-HCl pH 8 containing 0.5 M NaCl, 20 mM imidazole and EDTA-free protease inhibitor cocktail). Lysozyme was added to a final concentration of 0.2 μg/mL and the culture was incubated for 20 minutes at room temperature. After a freeze-thaw cycle, leupetin (0.5 μg/mL) and pepstatin (0.7 μg/mL) were added and cell debris were removed by centrifugation (16000 x *g* for 30 minutes at 4°C). The lysate was loaded on a HiTrap^TM^ TALON^®^ crude column equilibrated with 50 mM Tris-HCl pH 8 containing 500 mM NaCl and 20 mM imidazole. A linear gradient (0-100% in 100 ml) was then applied with the same buffer containing 500 mM imidazole. OxDC-His and OxDC DSSN-His eluted approximately at 200 mM imidazole. Upon forced dialysis by Amicon ultra 10 devices (10000 kDa) and wash with storage buffer (50 mM Tris-HCl pH 8, 500 mM NaCl and 5 mM DTT), OxDC-His and OxDC-DSSN-His can be conserved at -80°C where they are stable for several months. The purification efficiency was evaluated by SDS-PAGE: 1 μg of OxDC or OxDC-DSSN were loaded per lane on 10% polyacrilmide gel along with the Precision plus protein Kaleidoscope^TM^ (Bio-Rad) molecular mass markers. To remove the histidine tag, OxDC-His and OxDC-DSSN-His were incubated with 4 U of thrombin per mg of protein in 50 mM Tris-HCl pH 8, 500 mM NaCl, 5 mM dithiothreitol (DTT) buffer at 4°C for 16 hours with gentle mixing. The cleaved protein was then separated from thrombin excess by a Sephacryl S-300 column equilibrated and run in 50 mM Tris-HCl pH 8 containing 500 mM NaCl. Eluted proteins were concentrated to about 8 mg/ml using Amicon Ultra concentrators and stored at -80°C for at least 6 months.

***Decarboxylaseand oxidase activity assays***

The oxalate decarboxylase activity was determined by a coupled spectrophotometric assay that determines formate production by using formate dehydrogenase (FDH), which catalyses the oxidation of formate to carbon dioxide. The assay was carried out according to Magro *et al.* [1] with some modifications. During the purification procedure, the assay was performed in 52 mM Sodium Acetate pH 4.2 + 140 mM NaCl containing 63 mM potassium oxalate in the absence or presence of 0.5 mM *o*-Phenylenediamine (*o*-PDA). The buffer was chosen to keep constant the ionic strength to 154 mM, which represents a physiological value. The reaction was started by adding the enzyme (1 μg) and the mixture was incubated at 25°C for 4 minutes and stopped by the addition of 10 μl of NaOH 1M. Formate production was determined from the increase in the absorbance at 340 nm using FDH as described by Magro *et al.* [1].

To investigate the enzymatic activity in the pH range 4.2-7.2, we used the buffers reported in Supplementary Table 1. We maintained a constant NaCl concentration, while we changed the buffer concentration to keep a 154 mM ionic strength. The contribution of the stock enzyme to the final ionic strength of the mixture was also taken into account. At each pH, the potassium oxalate powder was dissolved in the assay buffer at 1 M concentration and the pH of each stock solution was checked. In order to evaluate possible buffer effects, buffer overlaps were performed at pH 5.5 and 7.0. Moreover, the reliability of the formate detection was evaluated at each pH by performing specific standard curves, as reported in Supplementary Fig. 1.

The decarboxylase specific activity (U/mg) was calculated by the equation:

$$AS=\frac{\left[ formate \left( \mu M \right)*\left( \frac{200 \mu l}{15\mu l} \right)*(0.11\mu l) \right]}{\min*mg of enzyme}$$

One unit of enzyme was defined as the amount of enzyme that produces 1 μmol of formate/min.

The oxalate oxidase activity was determined spectrophotometrically using a continuous assay in which the production of hydrogen peroxide (H_2_O_2_) was coupled to the oxidation of 2,2'-azino-bis(3-ethylbenzothiazoline-6-sulphonic acid (ABTS) by horseradish peroxidase (HRP) [2]. The oxidation of ABTS gives rise to a soluble end-product with an absorbance maximum at 420 nm (ε = 3.6 × 10^4^ M^–1^ cm^–1^). The reaction was carried out at physiological ionic strength using the same buffers and precautions described for the decarboxylase activity. The assay was started by adding 250 mM potassium oxalate to a mixture containing 5 μg of enzyme, 1 U of HRP, and 0.5 mM ABTS to a final volume of 250 μl. The increase in the absorbance at 420 nm was recorded by using a Jasco V-550 spectrophotometer with a temperature controlled cell-holder set at 25 °C. Blank sample, containing all components except the enzyme, were taken immediately prior to the measurements of samples containing protein. To validate the reliability of the oxidase assay in the 4.2-7.2 pH range, H_2_O_2_ solutions at known concentrations were assayed at each pH (data not shown).

The oxidase specific activity (U/mg) was calculated by the equation:

$$AS=\frac{\left[ \Delta Abs \left( {min}^{-1} \right)* {10}^{6}\left( M {min}^{-1} \right)*0.00025 L \right]}{\varepsilon_{ABTS}(M^{-1} \mathrm{cm}^{-1})*mg of enzyme}$$

One unit of enzyme was defined as the amount consuming or producing 1 µmol of substrate or product, respectively/min.

| ***pH*** | ***Buffer composition*** |
| --- | --- |
| 4.2 | 52 mM Sodium Acetate + 140 mM NaCl |
| 5.0 | 21 mM Sodium Acetate + 140 mM NaCl |
| 5.5 | 16.3 mM Sodium Acetate + 140 mM NaCl |
| 5.5 | 15.5 mM Bis-Tris + 140 mM NaCl |
| 6.0 | 18.5 mM Bis-Tris + 140 mM NaCl |
| 6.5 | 26 mM Bis-Tris + 140 mM NaCl |
| 7.0 | 58.3 mM Bis-Tris + 140 mM NaCl |
| 7.0 | 15 mM Tris-HCl + 140 mM NaCl |
| 7.2 | 16 mM Tris-HCl + 140 mM NaCl |

**Supporting Table 1**. Buffers used to study the effect of pH on OxDC and OxDC-DSSN activity


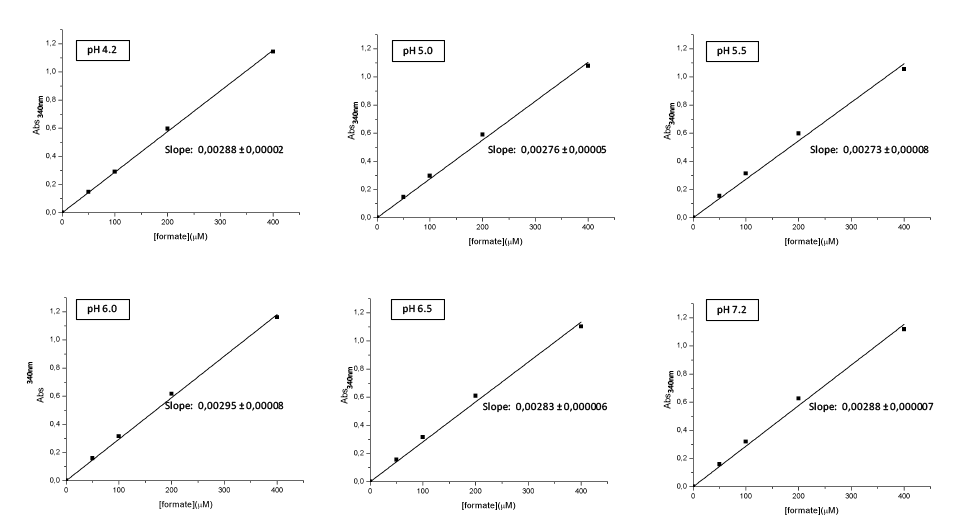


**Figure S1: FDH assay calibration curves.** Known formate concentrations (0, 50, 100, 200 and 400 μM) were dissolved in buffers reported in Supplementary Table 1 and detected using the FDH assay procedure. Data were fitted by linear regression and the resulting slope of each fitting is reported.

**Figure S2: Purification of OxDC and OxDC-DSSN.** (**A**) SDS-PAGE analysis of the various steps of OxDC-His and OxDC-DSSN-His purification. Lanes are coded as follows: 1 molecular weight markers; 2 and 5 soluble fraction of the cell lysate; 3 and 6 flow-through; 4 and 7 eluted OxDC-His and OxDC-DSSN-His, respectively. **(B**) Chromatographic profile of cleaved OxDC and OxDC-DSSN on a Sephacryl S-300 column. The buffer was 50 mM Tris- HCl pH 8 containing 500 mM NaCl. We checked by repeated injections that the peak present in the OxDC sample eluting before the void volume of the column (38 ml) is not an artefact and it is not due to previous injections. Although we do not know the origin of this species, we speculate that it possibly represents an high-MW OxDC aggregate that is excluded from the pores not by size but by charge-charge repulsion [3]. **(C)** SDS-PAGE analysis of OxDC-His (lane1), OxDC (lane 2), OxDC-DSSN-His (lane 3) and OxDC-DSSN (lane 4).

**Figure S3: Cross-linking analysis of OxDC and OxDC-DSSN.** Cross-linking of the purified proteins was performed using 1% glutaraldehyde; 7 μg of protein were loaded in SDS-PAGE. Lanes are coded as follows: 1, molecular weight markers; 2, OxDC; 3, OxDC-DSSN.

**Figure S4: Aggregation of OxDC and OxDC-DSSN at pH 4.2 and 7.2.** Time-dependent changes of the species present in solution (represented as size) of OxDC at pH 4.2 (A) and 7.2 (B) and OxDC-DSSN at pH 4.2 (C) and 7.2 (D). The coloured points represent the hexamers, while the black points represent aggregated species. All experiments were carried out at 0.5 mg/ml protein concentration in 52 mM sodium acetate pH 4.2, 140 mM NaCl or 16 mM Tris-HCl pH 7.2, 140 mM NaCl.

REFERENCES

1. Magro, P.M., P.; Di Lena P., *Enzymatic oxalate decarboxylation in isolates of Sclerotinia sclerotiorum.* FEMS Microbiology Letters, 1988. **49**(1): p. 49-52.

2. Requena, L. and S. Bornemann, *Barley (Hordeum vulgare) oxalate oxidase is a manganese-containing enzyme.* Biochem J, 1999. **343 Pt 1**: p. 185-90.

3. Asakawa, D., et al., *Optimization of conditions for high-performance size-exclusion chromatography of different soil humic acids.* Anal Sci, 2008. **24**(5): p. 607-13.
